# Differences in transcriptional changes in psoriasis and psoriatic arthritis skin with immunoglobulin gene enrichment in psoriatic arthritis

**DOI:** 10.1093/rheumatology/kead195

**Published:** 2023-05-03

**Authors:** Hanna Johnsson, John Cole, Iain B McInnes, Gerard Graham, Stefan Siebert

**Affiliations:** School of Infection and Immunity, University of Glasgow, Glasgow, UK; School of Infection and Immunity, University of Glasgow, Glasgow, UK; School of Infection and Immunity, University of Glasgow, Glasgow, UK; School of Infection and Immunity, University of Glasgow, Glasgow, UK; School of Infection and Immunity, University of Glasgow, Glasgow, UK

**Keywords:** psoriasis, PsA, inflammation, skin, immunoglobulins

## Abstract

**Objectives:**

Approximately 20% of people with psoriasis develop PsA. Although genetic, clinical and environmental risk factors have been identified, it is not known why some people with psoriasis develop PsA. The skin disease is traditionally considered the same in both. This study compares transcriptional changes in psoriasis and PsA skin for the first time.

**Methods:**

Skin biopsies were collected from healthy controls (HC), and uninvolved and lesional skin from patients with PsA. Bulk tissue sequencing was performed and analysed using the pipeline Searchlight 2.0. Transcriptional changes in PsA skin were compared with existing sequencing data from participants with psoriasis without PsA (GSE121212). Psoriasis and PsA datasets could not be directly compared as different analysis methods were used. Data from participants with PsA in the GSE121212 dataset were used for validation.

**Results:**

Skin samples from 9 participants with PsA and 9 HC were sequenced, analysed and compared with available transcriptomic data for 16 participants with psoriasis compared with 16 HC. Uninvolved skin in psoriasis shared transcriptional changes with lesional skin in psoriasis, but uninvolved skin in PsA did not. Most transcriptional changes in psoriasis and PsA lesional skin were shared, but immunoglobulin genes were upregulated in PsA lesional skin specifically. The transcription factor POU2F1, which regulates immunoglobulin gene expression, was enriched in PsA lesional skin. This was confirmed in the validation cohort.

**Conclusions:**

Immunoglobulin genes are upregulated in PsA but not in psoriasis skin lesions. This may have implications for the spread from the cutaneous compartment to other tissues.

Rheumatology key messages

- Immunoglobulin genes are upregulated in PsA skin lesions but not in psoriasis skin lesions.
- There are fewer transcriptional changes in PsA uninvolved skin than in psoriasis uninvolved skin.

## Introduction

Approximately 20% of people with the inflammatory skin condition psoriasis develop PsA [1]. Factors associated with the development of PsA include a family history of PsA, obesity and trauma [2]. People with more severe psoriasis, scalp psoriasis and psoriatic nail disease are at increased risk of developing PsA, but many only have mild skin disease. Clinically, the skin lesions in PsA and psoriasis have the same appearance, but a previous proteomic analysis of skin identified differentially expressed proteins in uninvolved and lesional skin in patients with psoriasis and PsA [3]. Previous skin transcriptomic studies have not distinguished between patients with and without psoriasis. Herein, we compare bulk tissue transcriptomic results in uninvolved and lesional skin in psoriasis and PsA with those in skin from healthy controls (HC) and use the results from these comparisons to identify differential gene expression patterns in psoriasis and PsA skin.

## Methods

### PsA and HC participants and samples

Paired biopsy samples of uninvolved and lesional skin from nine participants with PsA were collected and sequenced along with skin samples from nine HC (Johnsson *et al*. 2023 [4], GSE205748). Participants with PsA had active skin disease in an area acceptable to skin biopsy, and no participant received biologic medication. Briefly, 6 mm full-thickness skin biopsies were collected from within psoriasis lesions (1 cm from the edge) and from uninvolved skin (>8 cm from any lesion) from participants with PsA, and from the buttock area of HC participants. The project was reviewed and approved by the West of Scotland Research Ethics Committee (reference 16/WS/0059). All participants gave written informed consent before any study-related procedures.

### RNA extraction, cDNA library preparation and RNA sequencing

RNA was extracted from skin as per the RNeasy Mini RNA extraction kit (Qiagen) protocol and treated with DNase MAX (Qiagen). Polyadenylated RNA was selected using the NEBNext Poly(A) mRNA Magnetic Isolation Module kit (NEB #E7490), and the cDNA library was prepared using the NEBNext Ultra II Directional RNA Library Prep Kit with sample purification beads (NEB # E7765S). Sequencing was performed at Edinburgh Genomics (https://genomics.ed.ac.uk/) on the NovaSeq S1 (Illumina) using paired end sequencing of 50 base pairs with a total of 750M + 750M reads.

### RNA sequencing analysis

DESeq2 v1.24 was used to normalize the read counts and perform pairwise differential expression (PDE) analysis between HC and uninvolved skin, and HC and lesional skin (Supplementary Data S1, available at *Rheumatology* online). The automated pipeline Searchlight 2.0 was then used to aid the high-level analysis [5]. Genes with an absolute log2fold change >1 and adjusted *P*-value (*P*_adj_) < 0.05 were considered significantly differentially expressed. Searchlight 2.0 used the global gene expression data to generate principal component analysis (PCA) plots. Hypergeometric gene set enrichment analysis was performed to determine what biological processes differentially expressed genes (DEGs) were involved in (pathway analysis). This used pre-defined gene sets in the Gene Ontology database (http://geneontology.org/). The TRRUST (Transcriptional Regulatory Relationships Unravelled by Sentence-based Text mining) database was used to perform upstream regulator analysis. A hypergeometric gene set analysis was performed to identify significantly enriched upstream regulators and *P*_adj_ < 0.05 and absolute log2fold enrichment >0.0 was considered significant.

### Psoriasis and PsA validation cohorts

Bulk transcriptomic data from the biopsies of 16 participants with psoriasis without PsA in the publicly available dataset GSE121212 were downloaded from the gene expression omnibus database, together with data for 16 age- and gender-matched HC samples from the same study [6]. Transcriptomic data from four participants with PsA from the GSE121212 dataset were downloaded and compared with four matched HC samples. Each dataset was analysed separately using DESeq2 v1.24 and Searchlight 2.0, as above, comparing gene expression in the uninvolved and lesional skin to the respective HC group.

### Comparison of datasets and statistical analysis

Psoriasis and PsA samples could not be directly compared as these were different datasets generated using different methods. Therefore, patient samples were first compared with HC samples in the respective cohorts as outlined above (i.e. PsA *vs* HC; psoriasis *vs* HC; and Tsoi’s PsA validation *vs* HC), and then significant DEGs and enriched pathways were compared between cohorts.

Gene expression was converted to per-gene *z*-scores to enable comparison of genes with different levels of expression and to calculate the average expression values in individual samples. Spearman correlation coefficient was used to compare log2fold changes in gene expression and pathway enrichment between different analyses, and to correlate gene expression with disease characteristics. To compare clinical characteristic and the expression of specific genes, analysis of variance or Kruskal–Wallis test were used depending on the distribution of data.

## Results

### Participant characteristics

The participant characteristics are shown in Supplementary Table S1, available at *Rheumatology* online. Participants in the PsA group were older than participants in the other groups. However, the difference in ages was not significant (*P* = 0.3036). The skin disease was mild in both cohorts with median Psoriasis Area Severity Index (PASI) scores of 5.0 (interquartile range 3.5; 6.8) and 5.3 (interquartile range 5.2; 10.8) in the psoriasis and PsA cohorts, respectively. The participants in the psoriasis cohort and four participants in the PsA cohort were not receiving any systemic disease-modifying treatment. Three participants with PsA received MTX and two received apremilast.

### Psoriasis but not PsA uninvolved skin shares transcriptional changes with lesional skin

**Figure 1.**
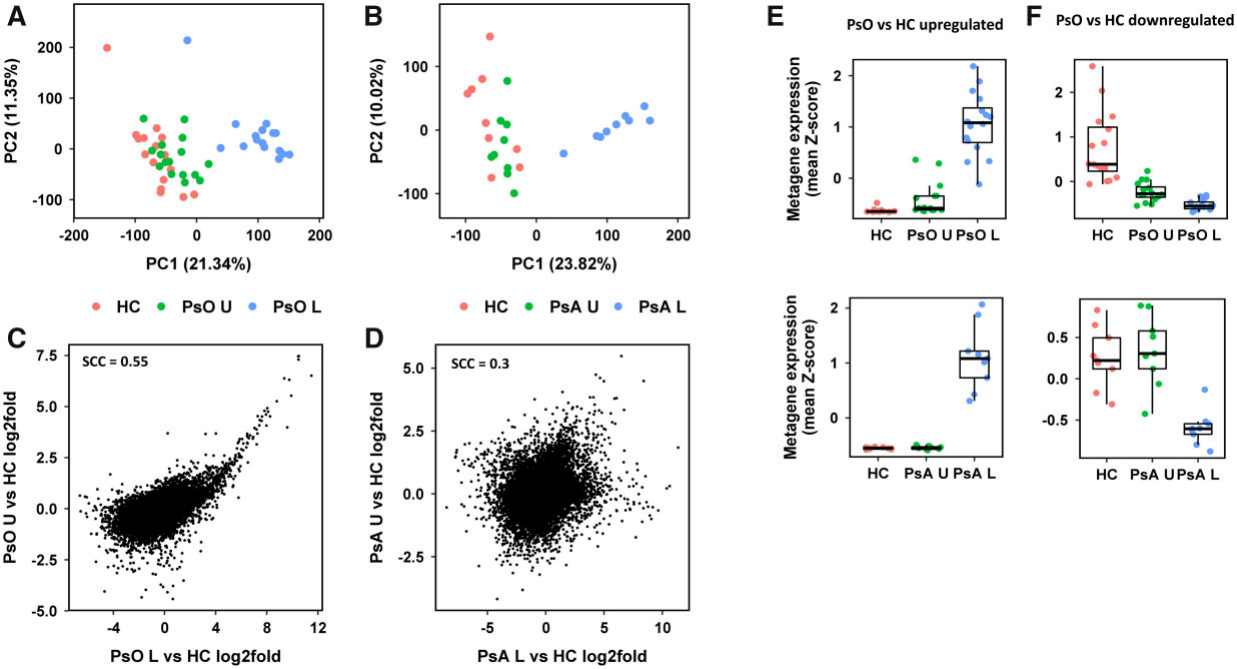
Similarities and differences in global gene expression. Principal component analysis (PCA) scatterplots for (**A**) the Tsoi psoriasis cohort and (**B**) our PsA cohort. Individual samples are represented by dots. The percentage of total variation explained by each component is given on the x- and y-axis. The scatterplots in (**C**) and (**D**) show the relationship between the log2fold changes for (C) psoriasis lesions (PsO L) *vs* healthy controls (HC) and psoriasis uninvolved (PsO U) *vs* HC and (D) PsA lesions (PsA L) *vs* HC and PsA uninvolved (PsA U) vs HC. Each dot represents the change in expression of one gene in the cohort, and the entire transcriptome is shown on each plot. The Spearman Correlation Coefficients (SCC) are given. (**E**) Expression boxplot showing the average expression of the 97 upregulated genes between PsO U and HC. Each dot represents a sample. The expression values were converted to per-gene *z*-scores before calculating the average expression of the 97 genes in the (top) psoriasis and (bottom) PsA cohorts. (**F**) As (E) but for the 27 downregulated genes between PsO U and HC

To gain an overview of differences in gene expression between the groups, and to visualize how transcriptionally similar, or different the samples were overall, PCA was performed for the psoriasis (Fig. 1A) and PsA (Fig. 1B) cohorts. The lesional samples (shown in blue) in each analysis grouped together indicating that the samples were transcriptionally similar and separate from the HC (red) samples. In contrast, the PsA uninvolved skin samples (green) grouped closely with the HC samples, while psoriasis uninvolved skin samples were also associated with the HC samples but skewed towards the psoriasis lesional skin group.

This suggests that there are transcriptional changes from HC in psoriasis uninvolved skin which are not present in PsA uninvolved skin. This was confirmed when the log2fold changes in gene expression in lesional and uninvolved skin compared with HC skin were compared. There was a positive correlation between DEGs in uninvolved and lesional skin in psoriasis compared with HC (Fig. 1C), but no correlation between the 15 DEGs in PsA uninvolved skin and those in PsA lesional skin compared with HC skin (Fig. 1D).

There were 124 DEGs in uninvolved psoriasis skin compared with HC skin, with 97 upregulated and 27 downregulated genes. Average expression of these DEGs showed a stepwise increase in expression of upregulated genes from HC through psoriasis uninvolved to psoriasis lesional skin (Fig. 1E, top panel), and a stepwise reduction in expression of downregulated genes (Fig. 1F, top panel). In contrast, there was no difference in the expression of these same genes in PsA uninvolved skin compared with HC skin (Fig. 1E and F, bottom panels). In PsA lesional skin, there was an increase in average expression of the genes which were upregulated in psoriasis uninvolved skin and reduced expression of downregulated genes.

**Figure 2.**
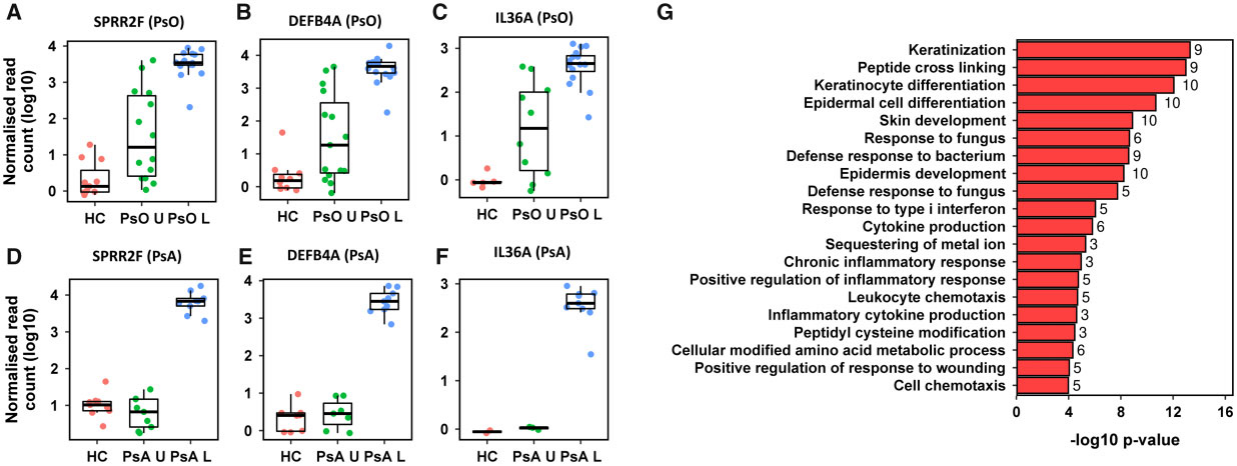
Upregulated genes and pathways in psoriasis uninvolved skin. The three most upregulated genes in psoriasis uninvolved compared with healthy control (HC) skin were (**A**) SPRR2F (log2fold 7.47, *P*_adj_ = 2.163e-13) (**B**) DEFB4A (log2fold 7.46, *P*_adj_ = 5.007e-12) and (**C**) IL-36A (log2fold 7.3, *P*_adj_ = 2.104e-9). Each dot represents gene expression in an individual sample. The normalized read count for the respective sample was plotted on a log10 axis and presented with medians and interquartile range. (**D**–**F**) The expression of the same genes in the PsA cohort. (**G**) Summary bar chart of the 20 most enriched genes sets (Gene Ontology, over-representation analysis, hypergeometric test) for significantly upregulated genes (*P*_adj_ < 0.05, absolute log2fold >1.0) between psoriasis uninvolved and HC skin. The enrichment adjusted *P*-value (–log_10_) are given on the x-axis and the gene set on the y-axis. The data labels denote the number of differentially expressed genes within each enriched gene set. All gene sets shown were significantly enriched (*P*_adj_ < 0.05)

The significantly upregulated DEGs in psoriasis uninvolved skin included immune and epidermal genes. The three most upregulated genes by log2fold change were SPRR2F (log2fold 7.47, *P*_adj_ = 2.163e-13), DEFB4A (log2fold 7.46, *P*_adj_ = 5.007e-12) and IL-36A (log2fold 7.3, *P*_adj_ = 2.104e-9) (Fig. 2A–C). These three genes were also significantly upregulated in psoriasis and PsA lesional skin but not in PsA uninvolved skin (Fig. 2D–F). Among the upregulated DEGs in uninvolved psoriasis skin, there were 21 enriched pathways (Fig. 2G), with keratinization as the most enriched pathway. There were no enriched pathways among the DEGs in PsA uninvolved skin.

**Figure 3.**
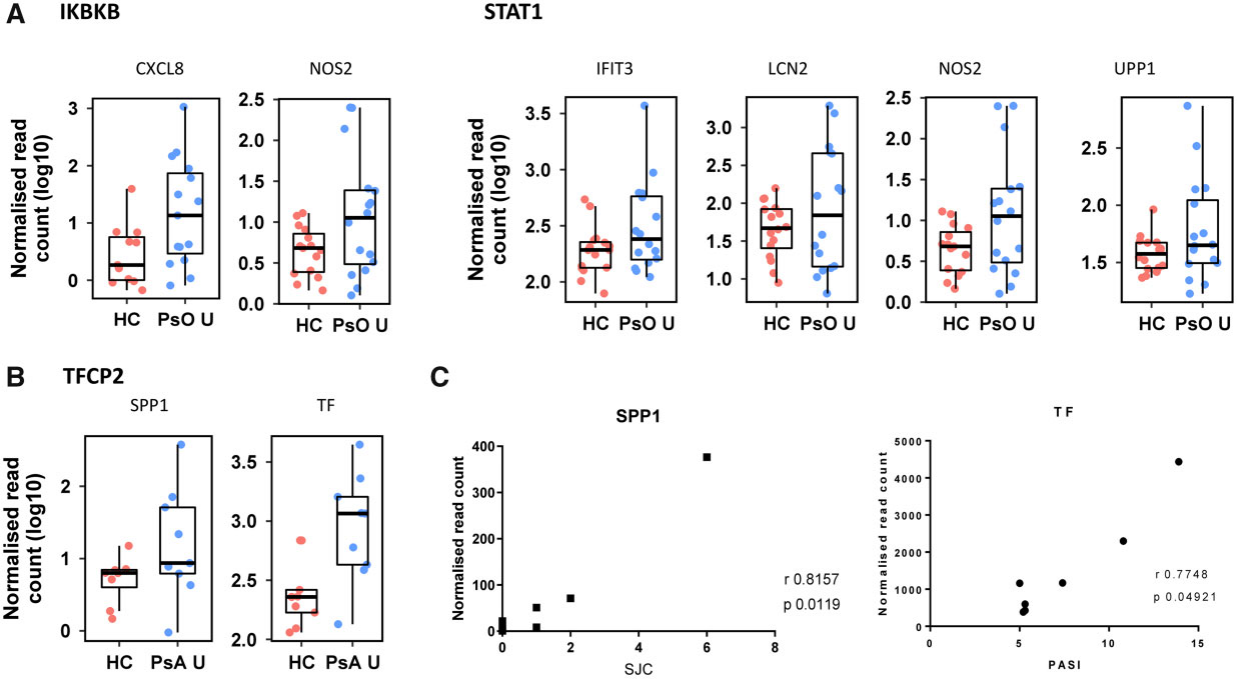
Enriched upstream regulators in uninvolved skin. (**A**) Summary boxplots of genes controlled by the enriched upstream regulators IKBKB and STAT1 in psoriasis uninvolved (PsO U) *vs* healthy control (HC) skin. (**B**) Summary boxplot of the genes regulated by the transcription factor TFCP2 and upregulated in PsA uninvolved (PsA U) compared with HC skin. Gene names are given on the x-axis and the expression values scaled into per-gene *z*-scores on the y-axis. (**C**) The expression of SPP1 in PsA uninvolved skin correlated with swollen joint count (SJC), and the expression of TF in PsA uninvolved skin correlated with the Psoriasis Area Severity Index (PASI) score

Next, we evaluated enriched upstream regulators in uninvolved skin. There was significant enrichment of genes controlled by the transcription factors IKBKB (log2fold enrichment 6.361, *P*_adj_ = 0.040) and STAT1 (log2fold enrichment 3.679, *P*_adj_ = 0.040, Fig. 3A) in psoriasis uninvolved skin. In PsA uninvolved skin, there was enrichment of the transcription factor CP2 (TFCP2) (log2fold enrichment 9.1489, *P*_adj_ = 0.0015). TFCP2 controls the expression of both SPP1 and TF, which were upregulated in uninvolved PsA skin compared with HC skin (Fig. 3B). SPP1 codes for the protein osteopontin which was also upregulated in PsA lesional skin (log2fold 2.32, *P*_adj_ = 0.0091). TF encodes transferrin which was unchanged in PsA lesional skin (log2fold 0.65, *P*_adj_ = 0.3285). In PsA uninvolved skin, the expression of SPP1 was positively correlated with swollen joint count, while the expression of TF correlated with PASI score (Fig. 3C).

### Psoriasis and PsA lesional skin share transcriptional changes and pathway enrichments

**Figure 4.**
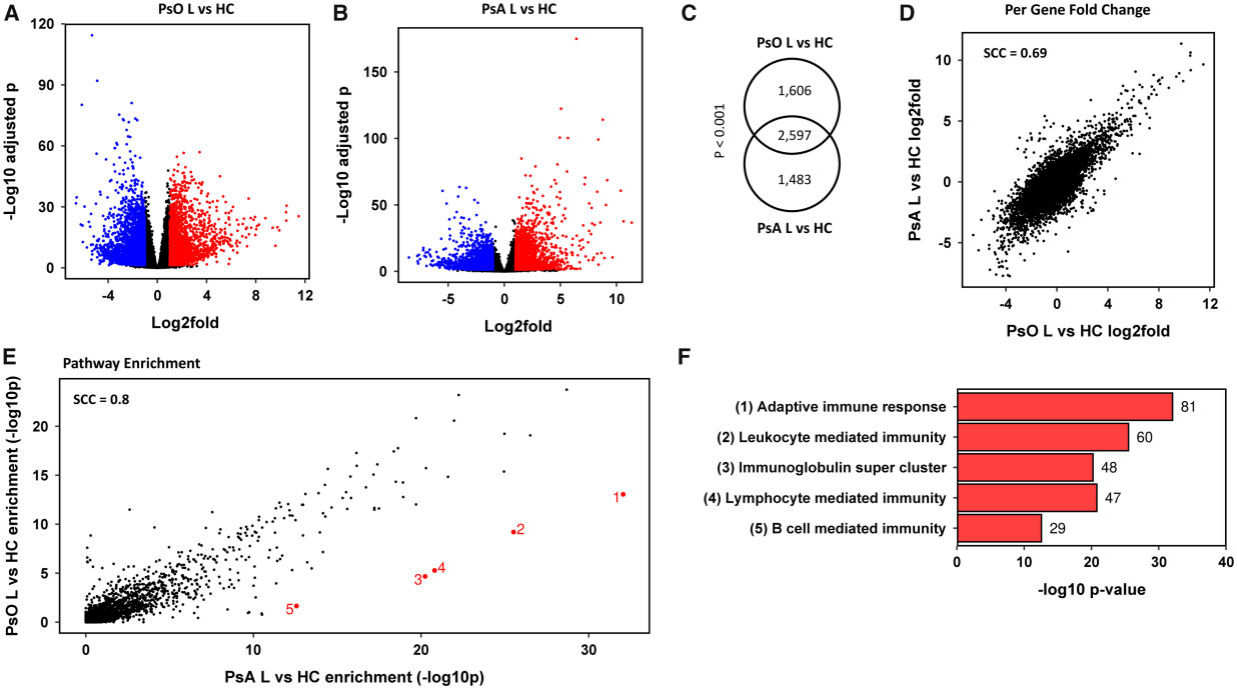
Significant DEGs in psoriasis and PsA lesional skin. Volcano plots for the comparisons between psoriasis lesional (PsO L) and healthy control (HC) skin (**A**) and PsA lesional (PsA L) and HC skin (**B**). Significant DEGs (*P*_adj_ < 0.05, absolute log2fold >1) are shown on the right in red for upregulated genes, on the left in blue for downregulated genes and non-significant genes in the middle in black. A positive fold change indicates higher expression in skin lesions than in HC. (**C**) Venn diagram showing the overlap between DEGs of PsO L *vs* HC and PsA L *vs* HC. The *P*-value (hypergeometric test) is given. (**D**) Relationship between the log2fold changes for PsO L *vs* HC and PsA L *vs* HC. Each dot represents one gene, and the entire transcriptome is shown. The Spearman Correlation Coefficient (SCC) is given. (**E**) Pathway enrichment in lesional skin showing the relationship between Gene Ontology enrichment (–log10 enrichment *P*-value) for PsO L *vs* HC and PsA L *vs* HC. Each dot represents one gene ontology. All ontologies are shown. The five ontologies with the largest absolute difference in enrichment are numbered, with data labels indicating the rank. (**F**) Bar chart showing the five ontologies with the largest absolute difference in enrichment from (E). The –log10 enrichment *P*-value is given on the x-axis and the gene set name on the y-axis. Data labels represent the number of significant differentially expressed genes within each gene set. The gene sets shown were significantly enriched (*P*_adj_ < 0.05, absolute log2fold >0.0). Enrichment analysis was performed using Hypergeometric Gene Set Enrichment on the gene set databases Gene Ontology Biological Process

There were >6000 significant DEGs when psoriasis and PsA skin lesions were compared with their respective HC groups. There were large numbers of both upregulated and downregulated DEGs as shown in the volcano plots in Fig. 4A and B. Most significant DEGs were shared (Fig. 4C), with a positive correlation between the significant DEGs in the two lesional skin cohorts (Fig. 4D), implying that the same genes were DEGs in psoriasis and PsA skin lesions.

Pathway analysis identified 13 enriched pathways among downregulated genes in psoriasis lesions and 19 in PsA lesions (Supplementary Fig. S1, available at *Rheumatology* online). Pathways relating to muscle contraction and neuronal synapse were present in both analyses. The ‘neutral lipid metabolic process’ was enriched in PsA lesions only but most genes within the gene set were downregulated in psoriasis lesions also.

There were more enriched pathways among upregulated genes in lesional skin, with enrichment of 308 pathways in psoriasis lesions and 662 pathways in PsA lesions compared with their respective HC groups. There was a strong correlation between the pathway enrichment in psoriasis and PsA lesions with a Spearman correlation coefficient of 0.8 (Fig. 4E). However, differences in pathway enrichment were observed, with the five pathways with the largest difference in enrichment shown in Fig. 4E. These pathways relate to the adaptive immune system, with differential enrichment of immunoglobulin and B cell–related immunity pathways (Fig. 4F, Supplementary Fig. S2, available at *Rheumatology* online). The pathway ‘B cell mediated immunity’ was significantly enriched in PsA lesions (log2fold enrichment 0.9671, *P*_adj_ = 0.002), but not in psoriasis lesions (log2fold enrichment 0.9825, *P*_adj_ = 0.1322).

### The expression of immunoglobulin genes is increased in PsA skin lesions

**Figure 5.**
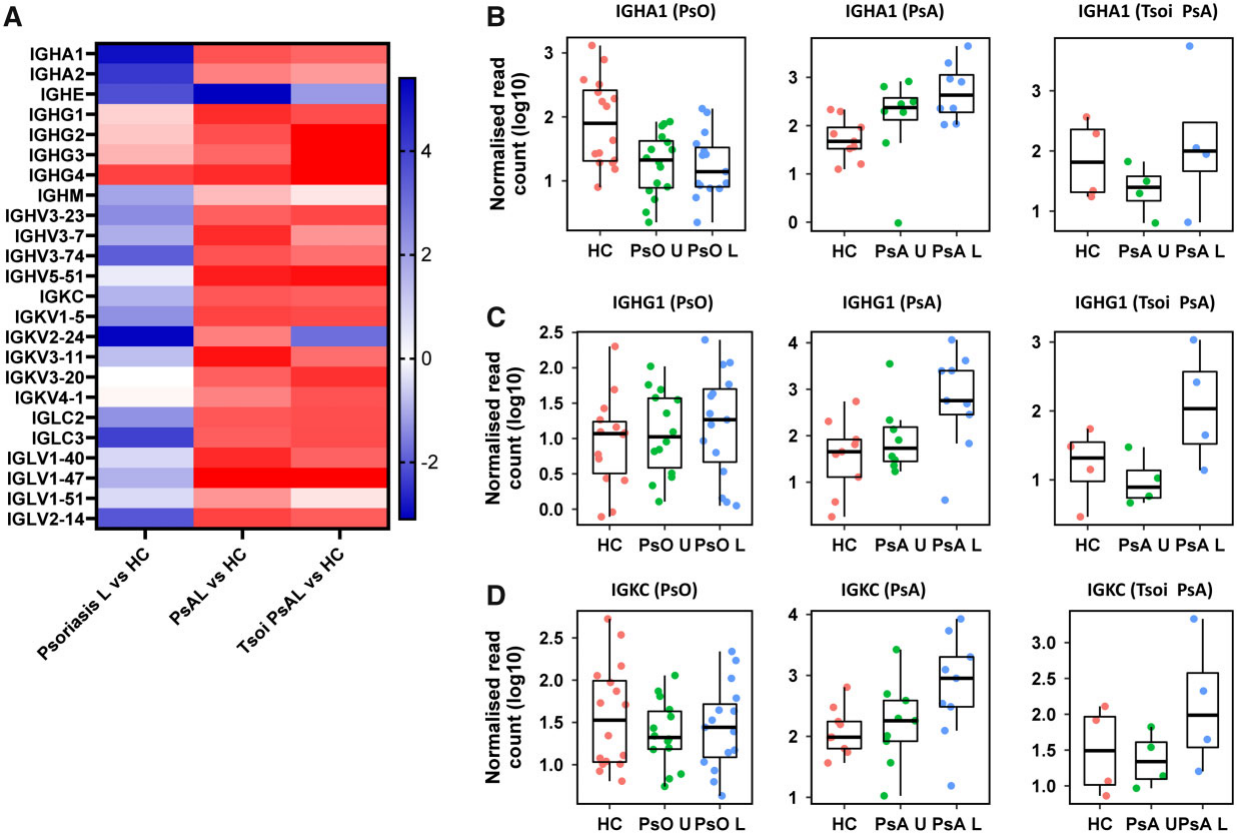
Immunoglobulin gene expression. (**A**) Heatmap showing the log2fold change in gene expression of the immunoglobulin genes on the y-axis for the comparisons with healthy controls (HC) for the Tsoi psoriasis lesional (psoriasis L) cohort, our PsA lesional (PsA L) cohort and a PsA validation cohort (Tsoi PsA L). Red indicates upregulation and blue downregulation in skin lesions compared with HC. The normalized, log10-transformed read counts in individual samples in each cohort were plotted for immunoglobulin genes IGHA (**B**), IGHG1 (**C**) and IGKC (**D**). Each dot represents one sample and median and interquartile ranges are indicated. PsO U: psoriasis uninvolved; PsO L: psoriasis lesional; PsA U: psoriasis uninvolved

The DEGs responsible for this pathway enrichment in PsA skin lesions were largely upregulated immunoglobulin genes (Supplementary Table S2, available at *Rheumatology* online). A heatmap of the log2fold changes in immunoglobulin gene expression in skin lesions in psoriasis and PsA compared with HC is shown in Fig. 5A. All immunoglobulin genes shown were significantly upregulated in PsA lesions compared with HC, apart from IGHM, which was unchanged, and the heavy constant chain of IgE (IGHE), which was downregulated. The latter was also downregulated in psoriasis lesions. In contrast, most immunoglobulin genes in psoriasis lesions were unchanged or downregulated, and only the IgG heavy constant IGHG4 was significantly upregulated in psoriasis lesions compared with HC.

The Tsoi GSE121212 dataset included data from four participants with PsA, which were analysed separately as a validation cohort. The changes in immunoglobulin gene expression in these samples confirmed the consistent upregulation of immunoglobulin genes in our PsA skin lesions compared with HC (right column in heatmap, Fig. 5A).

The expression of immunoglobulin genes varied between genes and samples. The genes coding for the heavy constants of alpha 1 (IGHA1) and gamma 1 (IGHG1), and the kappa constant of the light chain (IGKC) had the highest normalized read counts. The expression values in individual samples for these three genes are shown in Fig. 5B–D, demonstrating that although there was heterogeneity between samples, there was increased expression of immunoglobulins in most PsA skin lesions in both PsA lesional cohorts.

### Among predicted upstream regulators, POU2F1 was specifically enriched in PsA skin lesions

**Figure 6.**
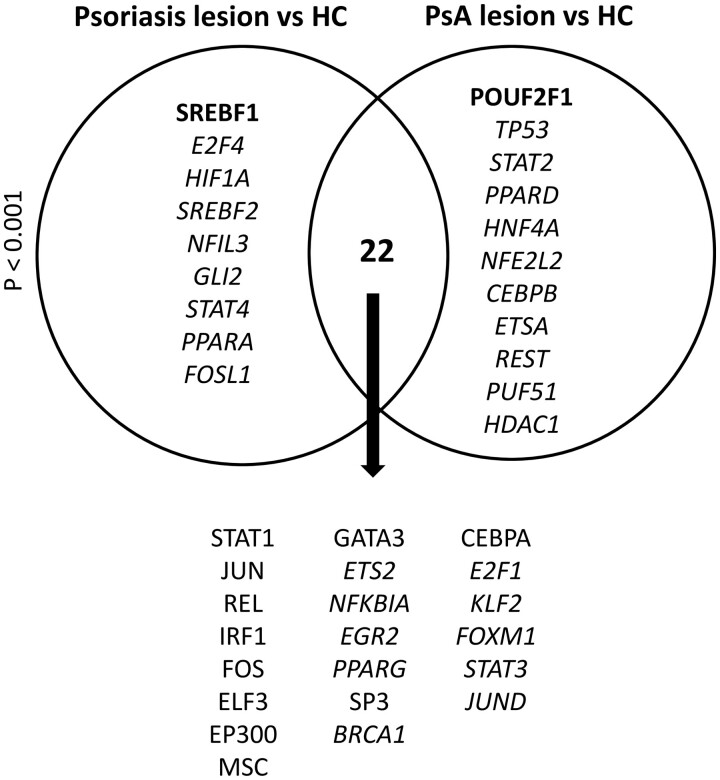
Enriched upstream regulators in psoriasis and PsA skin lesions. The significantly enriched upstream regulators identified by TRRUST (Transcriptional Regulatory Relationships Unravelled by Sentence-based Text mining) analysis are shown. Transcription factors enriched in psoriasis or PsA lesions only are given in the circles, with shared enriched transcription factors listed below the diagram. The transcription factors in bold were among the 10 most significantly enriched upstream regulators in one comparison but were not significantly enriched in the other comparison. The *P*-value for the overlap (hypergeometric test) is given. HC: healthy control

TRRUST analysis indicated that most significantly enriched upstream regulators were the same in psoriasis and PsA skin lesions (Fig. 6). Of the 10 most significantly enriched upstream regulators in each comparison, Sterol Regulatory Element Binding Transcription Factor 1 (SREBF1) was predicted to be enriched only in psoriasis lesions, and POU class 2 homeobox 1 (POU2F1) only in PsA lesions. POU2F1 was also significantly enriched in PsA lesional skin in the validation cohort.

## Discussion

This study compared whole skin transcriptional changes in lesional and uninvolved skin in psoriasis and PsA skin for the first time. The main finding was upregulation of immunoglobulin genes in PsA skin lesions but not in psoriasis skin lesions. This was also observed in a separate small PsA validation cohort. Moreover, the transcription factor POU2F1 was specifically enriched in PsA skin lesions. POU2F1, although not specific for B cells, interacts with the octamer element, a motif conserved within Ig promoters and enhancers, and mediates transcription from the Ig loci [7]. It is thus possible that increased POU2F1 activity contributes to the upregulation of immunoglobulin genes in PsA skin lesions.

The differential upregulation of immunoglobulin genes is a novel finding and raises the possibility of different immune mechanisms in the cutaneous inflammation in psoriasis and PsA, with a distinct role of immunoglobulins in PsA skin. Psoriatic disease is traditionally considered a T cell–mediated disease, but these findings also suggest involvement of the B cell compartment in lesional skin in patients with PsA. B cells in skin have not been studied as extensively as T cells, but there is increasing evidence of B cell subsets and antibody secreting cells playing a role in maintaining skin homeostasis [8]. For example, secretory IgM and IgA are produced by normal skin and bind to commensals and invading microbes, B cells support wound healing and regulatory B cells (Bregs) produce IL-10, which limits inflammation. Circulating Bregs are reduced in people with psoriasis and PsA but increase following treatment with apremilast and correlate with skin and joint improvement in response to apremilast [9].

Others have observed a small number of B cells in a majority (14/15) of PsA skin lesions, but only in a minority (1/5) of psoriasis lesions [10], while plasma cells were reported in 21% of patients with psoriasis with unknown PsA status [11]. In PsA synovium, CD20^+^ B cells were found at similar rates to seronegative RA on immunohistochemistry, but CD168^+^ plasma cells were only rarely seen [12]. Lymphoid aggregates were however found in most PsA joints, with ectopic lymphoid neogenesis features present in half [13]. Interestingly, recent single cell analysis of synovium from patients with PsA and RA did not identify a difference in the relative abundance of four B cell subsets between PsA and RA, but found differences in B cell–pathway enrichment which requires further investigation [14].

Further evidence for a role of the B cell compartment in psoriatic disease include an increase in circulating antibodies, specifically against LL-37 and ADAMTS-L5 [15]. These antibodies are found in higher concentrations in patients with PsA than those with psoriasis alone and correlate with joint disease severity [15, 16]. LL-37 auto-antibodies are also found in PsA SF [16]. LL-37 is an antimicrobial peptide (cathelicidin) and ADAMTS-L5 promotes fibril formation in the extracellular matrix. Both antigens are found in the skin and are upregulated in psoriatic skin lesions where LL-37-specific cytokine-producing T cells have been reported [17, 18]. Moreover, LL-37 is overexpressed in PsA synovium where it co-localizes with IgG immune complexes [16].

This study has also demonstrated that changes in gene expression in uninvolved skin in psoriasis and PsA are subtly different, with fewer DEGs in PsA uninvolved skin compared with HC skin. In keeping with previous studies, uninvolved skin in psoriasis shared some transcriptional changes with psoriasis lesional skin, including upregulation of genes relating to keratinocyte differentiation and immune functions such as cytokine production, chronic inflammatory response and response to bacteria and fungi [19–21].

One factor which could potentially be contributing to the differences seen in uninvolved skin is the use of systemic treatment. Five PsA participants received systemic treatment at the time of biopsy while no psoriasis participants did. Prior systemic treatments in both cohorts are unknown. A further possibility is that differences in the microbiome affect the transcriptome, with a significantly higher abundance of *Atopobium* and *Megasphaera* reported in psoriasis uninvolved skin than in PsA uninvolved skin [22].

No significant pathway was enriched in uninvolved PsA skin, but two of the 15 significant DEGs (SPP1 and TF) are controlled by TFCP2, which is an ubiquitously expressed transcription factor with roles in regulating the cell cycle [23]. TFCP2 is implicated in repair processes with increased expression in regenerated cartilage [24], while related transcription factors regulate wound healing in *Drosophila* and mice [25, 26]. Interestingly, serum levels of SPP1, in conjunction with other proteins, were recently identified by machine learning as potential biomarkers to distinguish patients with PsA from those with psoriasis alone [27]. The presence of DEGs controlled by TFCP2 in the skin in PsA and association with serum levels raises the possibility that TFCP2 plays a role in the transition from cutaneous psoriasis to PsA.

The major limitation of this study is that a direct comparison between gene expression in psoriasis and PsA was not possible as different datasets were analysed. However, it enabled us to use the same method to analyse a PsA validation cohort. PsA is a heterogeneous disease and although the validation cohort had a low sample number, it supported our main findings. Another limitation is that five participants in the PsA cohort received systemic disease-modifying treatment and the effects of these are unknown. The differences observed do not appear to be due to worse skin disease in either group, as PASI scores were similar in the psoriasis and PsA cohorts. A further limitation is that no functional inferences can be made, and we have not confirmed which cell types are responsible for the increased expression of immunoglobulins. It cannot be concluded whether the increase in immunoglobulin expression is a cause or a consequence of the development of PsA.

In conclusion, the transcriptomes of psoriasis and PsA skin are not identical, and the increased expression of immunoglobulin genes in PsA skin lesions adds compelling evidence to the emerging literature for a potential role of the B cell compartment in psoriatic disease and PsA in particular. Future studies are required to determine whether this differential immune response within the skin lesions in PsA reflects underlying generation of immunoglobulins and plays a functional role in the spread of inflammation from the skin to joints in PsA.

## Supplementary Material

kead195_Supplementary_Data

## Data Availability

Data analysed during the current study are available in the Gene Expression Omnibus (GEO) repository, under accession GSE205748; https://www.ncbi.nlm.nih.gov/geo/query/acc.cgi?acc=GSE205748.
