# Supplementary material for: Differences in transcriptional changes in psoriasis and psoriatic arthritis skin with immunoglobulin gene enrichment in psoriatic arthritis
Source: Rheumatology (Oxford). 2023 May 3;63(1):218–25. doi: 10.1093/rheumatology/kead195 (PMC10765156; doi:10.1093/rheumatology/kead195)
Supplement: kead195_Supplementary_Data [file kead195_supplementary_data.zip › kead195_Supplementary_Data/rhe-23-0224-File008.docx]

## **Supplementary Data S1. Supplementary Methods**

## **RNA sequencing analysis**

To process raw RNAseq datasets we used the following pipeline: Firstly, the FastQ files were QC’d using FastQC v0.11.7 (1), and then were aligned to the reference genome using STAR v2.6 (2) with –quantMode GeneCounts –outFilterMultimapNmax 1 and –outFilterMatchNmin 35. We used a Star index with a –sjdbOverhang of the maximum read length − 1. Next, read count files were merged and genes with mean of < 1 read per sample were excluded. Finally, the expression and differential expression values were generated using DESeq2 v1.24 (3). For differential comparisons we used an A versus B model with no additional covariates. All other parameters were left to default. In short, DESeq2 v1.24 assessed the logarithmic fold change of gene expression in two groups (pairwise differential expression, PDE) and determined significance using the Wald test. Next, a filtering step excluded genes with little or no change of being detected as differentially expressed (valid or invalid PDE). Finally, Benjamini-Hochberg procedure was applied to control the false discovery rate of genes with a valid PDE. Sequences were aligned to the genome and transcriptome GRCh38 (release 91).

The data was explored and visualised using Searchlight2 (4). Specifying 2 differential expression workflows for each comparison (Lesion vs HC and Uninvolved vs HC) and one multiple differential expression workflow (Lesion vs HC + Uninvolved vs HC). The significance threshold for genes was set to p_adj_ = 0.05 and log2fold = 1. Over representation analysis was included using the default human GO biological processes database (5), and a threshold for significant enrichment of p_adj_ = 0.05. All other parameters were left to default.

1. FastQC. [Available from: <https://www.bioinformatics.babraham.ac.uk/projects/fastqc/>.

2. Dobin A, Davis CA, Schlesinger F, Drenkow J, Zaleski C, Jha S, et al. STAR: ultrafast universal RNA-seq aligner. Bioinformatics. 2013;29(1):15-21.

3. Love MI, Huber W, Anders S. Moderated estimation of fold change and dispersion for RNA-seq data with DESeq2. Genome Biol. 2014;15(12):550.

4. Cole JJ, Faydaci BA, McGuinness D, Shaw R, Maciewicz RA, Robertson NA, et al. Searchlight: automated bulk RNA-seq exploration and visualisation using dynamically generated R scripts. BMC Bioinformatics. 2021;22(1):411.

5. Ashburner M, Ball CA, Blake JA, Botstein D, Butler H, Cherry JM, et al. Gene ontology: tool for the unification of biology. The Gene Ontology Consortium. Nat Genet. 2000;25(1):25-9.

**Supplementary Table S1. Participant characteristics**

|  | **PsA**  **(n=9)** | **HC**  **(n=9)** | **Tsoi PsO (n=16)** | **Tsoi HC (n=16)** | **Tsoi PsA**  **(n=4)** | **Tsoi HC**  **(n=4)** |
| --- | --- | --- | --- | --- | --- | --- |
| **Age (years)** *mean*  (95% CI) | 46.4  (33.9; 59.0) | 36.4  (29.6; 43.3) | 37.4  (30.2; 44.7) | 37.1  (30.3; 43.9) | 50.5  (22.4; 78.6) | 48.8  (25.9; 71.6) |
| **Female** gender (%) | 5 (55.6%) | 6 (66.7%) | 6 (37.5%) | 6 (37.5%) | 2 (50%) | 2 (50%) |
| **PASI** *median*  (25^th^; 75^th^)* | 5.3  (5.2; 10.8) | N/A | 5.0  (3.5; 6.8) | N/A | 6.0  (4.0; 7.8) | N/A |
| **DMARD**  *Methotrexate*  *Apremilast* | n = 5  *n = 3*  *n = 2* | N/A | n = 0 | N/A | n = 0 | N/A |

PASI psoriasis area severity index *Data missing for 2 participants with psoriasis body surface area = 3%

**Supplementary Table S2. B cell mediated immunity pathway genes**

|  |  | **PsA L v HC** | | **Psoriasis L v HC** | |
| --- | --- | --- | --- | --- | --- |
| **Gene** | **Gene description** | **log2fold** | **p_adj_** | **log2fold** | **p_adj_** |
| BATF | basic leucine zipper ATF-like transcription factor | 1.3 | 8.76E-06 | 1.27 | 1.19E-06 |
| BCL3 | B-cell CLL/lymphoma 3 | 1.52 | 2.07E-21 | 1.51 | 2.65E-20 |
| C1QB | complement C1q B chain | 1.37 | 0.009726 | 1.07 | 0.023255 |
| C1QC | complement C1q C chain | 1.06 | 0.008547 | 0.73 | 0.067218 |
| C4BPB | complement component 4 binding protein beta | -1.69 | 0.004731 | -1.37 | 0.117966 |
| C6 | complement C6 | -1.9 | 0.001515 | -2.95 | 2.7E-06 |
| C7 | complement C7 | -1.1 | 0.0069 | -2.02 | 2.15E-06 |
| C9 | complement C9 | 2.77 | 6.5E-09 | 1.26 | 0.015094 |
| CD27 | CD27 molecule | 1.03 | 0.0077 | 0.61 | 0.088676 |
| CD40LG | CD40 ligand | 1.34 | 0.002712 | 0.79 | 0.024535 |
| CR1 | complement C3b/C4b receptor 1 | 1.29 | 0.031368 | -0.09 | 0.860331 |
| EXO1 | exonuclease 1 | 1.96 | 1.05E-13 | 1.29 | 2.46E-19 |
| IGHA1 | immunoglobulin heavy constant alpha 1 | 3.63 | 0.000805 | -2.92 | 5.19E-06 |
| IGHA2 | immunoglobulin heavy constant alpha 2 | 2.67 | 0.008475 | -2.43 | 0.001697 |
| IGHE | immunoglobulin heavy constant epsilon | -3.1 | 0.032423 | -2.15 | 0.071652 |
| IGHG1 | immunoglobulin heavy constant gamma 1 | 4.46 | 0.00037 | 0.94 | 0.313112 |
| IGHG2 | immunoglobulin heavy constant gamma 2 | 3.71 | 0.002217 | 1.19 | 0.298378 |
| IGHG3 | immunoglobulin heavy constant gamma 3 | 3.24 | 0.007304 | 1.58 | 0.097165 |
| IGHG4 | immunoglobulin heavy constant gamma 4 | 4.48 | 0.003944 | 4.02 | 0.019944 |
| IGHV3-23 | immunoglobulin heavy variable 3-23 | 3.38 | 0.011576 | -1.41 | 0.224164 |
| IGKC | immunoglobulin kappa constant | 3.54 | 0.000174 | -0.93 | 0.218357 |
| IGKV1-5 | immunoglobulin kappa variable 1-5 | 3.99 | 0.002427 | -1.34 | 0.253388 |
| IGKV3-20 | immunoglobulin kappa variable 3-20 | 3.37 | 0.008967 | -0.01 | 0.995526 |
| IGKV4-1 | immunoglobulin kappa variable 4-1 | 2.63 | 0.045005 | 0.17 | 0.889939 |
| IGLC2 | immunoglobulin lambda constant 2 | 3.55 | 0.004406 | -1.33 | 0.16901 |
| IGLC3 | immunoglobulin lambda constant 3 | 3.39 | 0.010819 | -2.32 | 0.028703 |
| IGLC7 | immunoglobulin lambda constant 7 | 4.81 | 0.032557 | not in dataset | |
| IGLL5 | immunoglobulin lambda like polypeptide 5 | 2.79 | 0.034801 | not in dataset | |
| IL4R | interleukin 4 receptor | 2.21 | 6.9E-48 | 2.26 | 1.2E-29 |
| IRF7 | interferon regulatory factor 7 | 2.23 | 1.77E-20 | 2.31 | 2.27E-23 |
| MASP2 | mannan binding lectin serine peptidase 2 | -1.24 | 9.18E-05 | -0.66 | 0.002474 |
| RNF168 | ring finger protein 168 | 1.02 | 1.27E-18 | 0.78 | 2.96E-24 |
| SLA2 | Src like adaptor 2 | 1.31 | 4.02E-07 | 1.08 | 3.73E-06 |
| SUSD4 | sushi domain containing 4 | 0.87 | 0.005783 | 1.72 | 2.42E-12 |
| TLR8 | toll like receptor 8 | 1.88 | 2.25E-05 | 1.12 | 0.000312 |
