# Supplementary figures and images for: Differences in transcriptional changes in psoriasis and psoriatic arthritis skin with immunoglobulin gene enrichment in psoriatic arthritis
Source: Rheumatology (Oxford). 2023 May 3;63(1):218–25. doi: 10.1093/rheumatology/kead195 (PMC10765156; doi:10.1093/rheumatology/kead195)

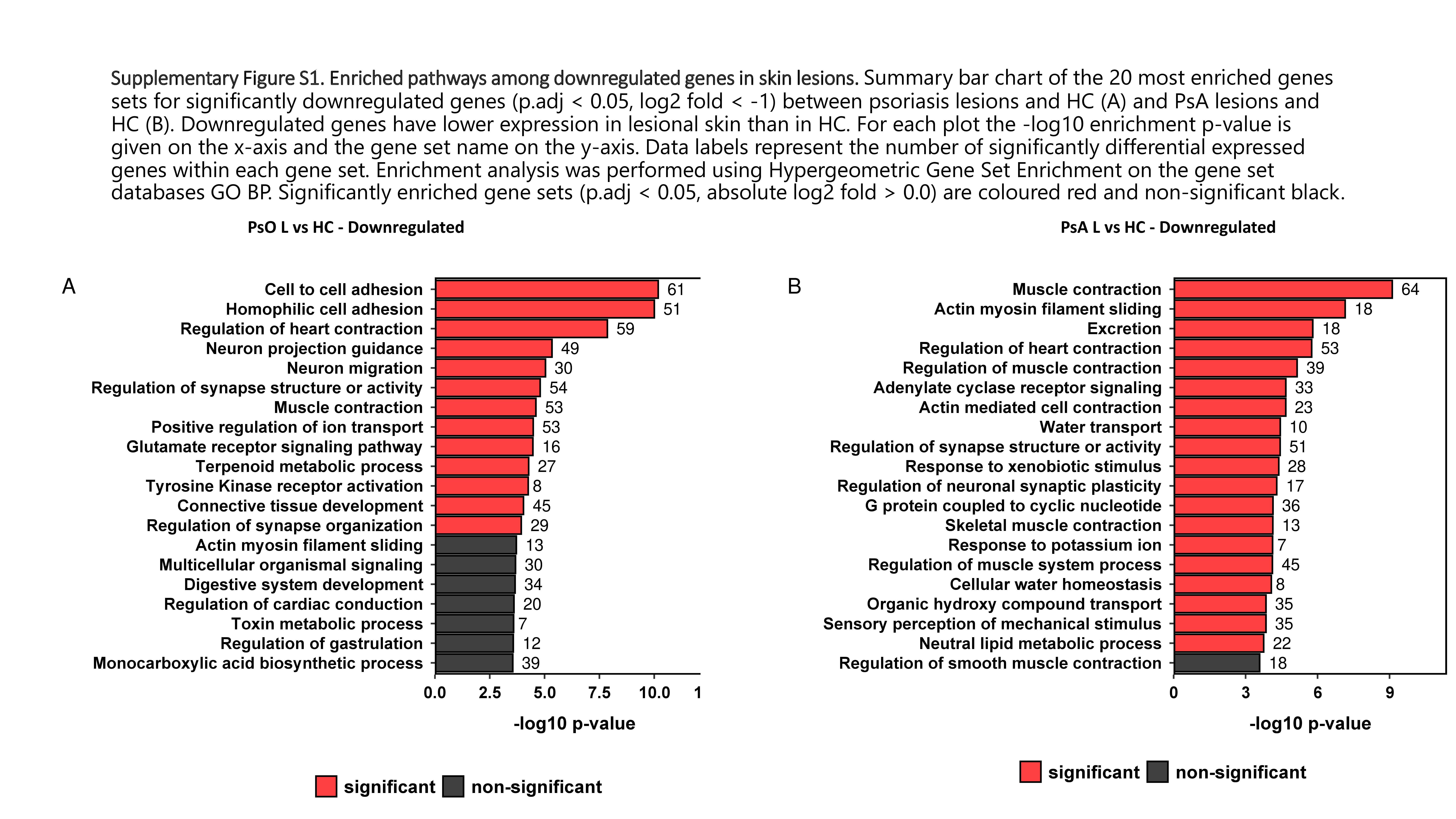

Supplement: kead195_Supplementary_Data [file kead195_supplementary_data.zip › kead195_Supplementary_Data/rhe-23-0224-File009.tiff]

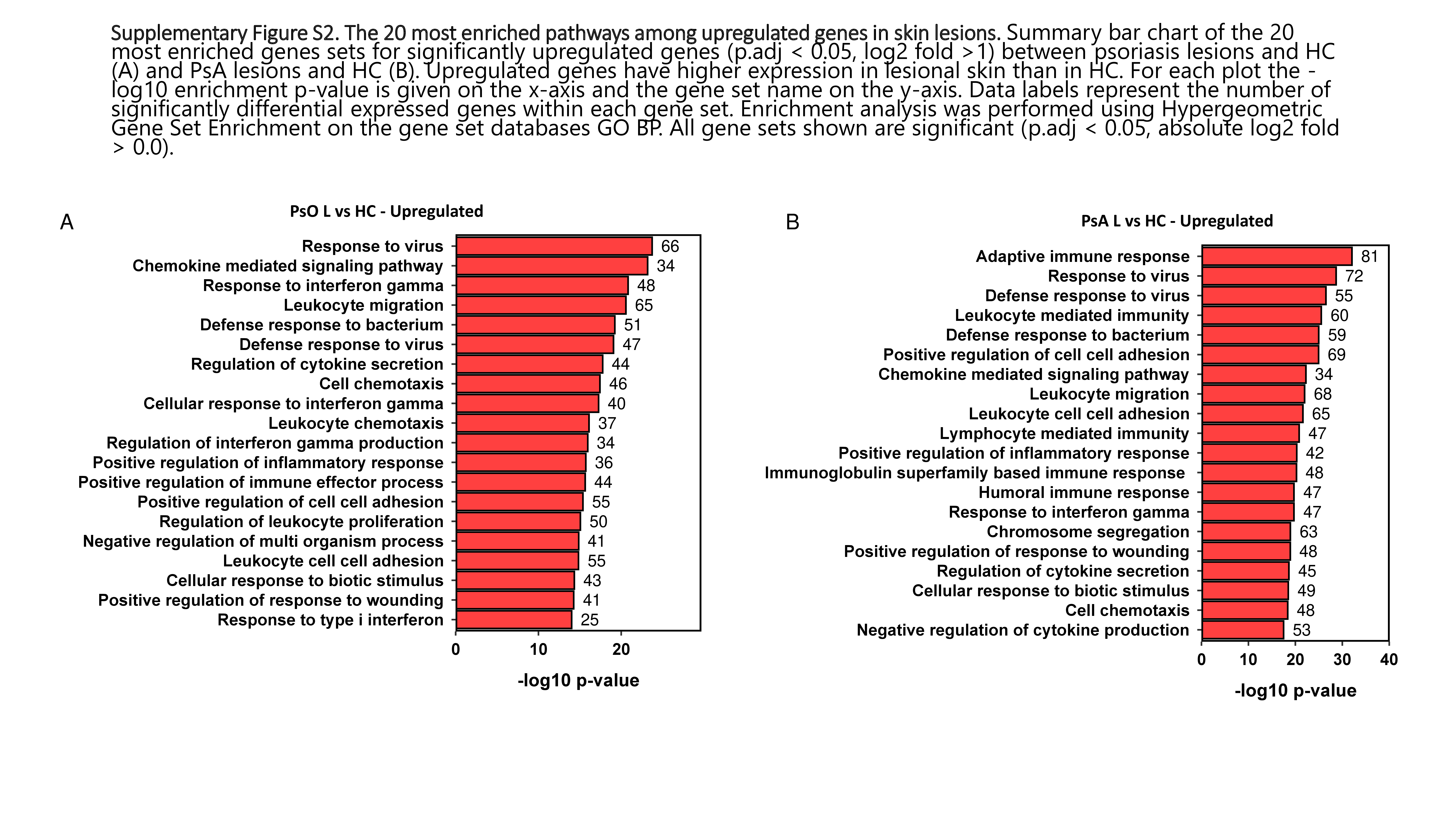

Supplement: kead195_Supplementary_Data [file kead195_supplementary_data.zip › kead195_Supplementary_Data/rhe-23-0224-File010.tiff]
